# Effects of virtual reality on spatiotemporal gait parameters and freezing of gait in Parkinson’s disease

**DOI:** 10.1038/s41531-025-01017-9

**Published:** 2025-06-04

**Authors:** Lei Ma, Bar Yosef, Ipek Talu, Daniel Batista, Emi Jenkens-Drake, Nanthia Suthana, Katy A Cross

**Affiliations:** 1 Department of Neurology, David Geffen School of Medicine, University of California Los Angeles, Los Angeles, CA USA; 2 Department of Psychiatry & Biobehavioral Sciences, Jane and Terry Semel Institute for Neuroscience and Human Behavior, University of California Los Angeles, Los Angeles, CA USA; 3 Department of Bioengineering, University of California Los Angeles, Los Angeles, CA USA; 4 Department of Neurosurgery, David Geffen School of Medicine, University of California Los Angeles, Los Angeles, CA USA; 5 Department of Psychology, University of California Los Angeles, Los Angeles, CA USA

**Keywords:** Parkinson's disease, Parkinson's disease, Neurological manifestations

## Abstract

Virtual reality (VR) is increasingly used to study freezing of gait (FOG) in Parkinson’s disease (PD). However, overground gait in VR typically exhibits shorter, wider, and slower steps than real-world gait in both healthy and PD populations. This altered gait behavior raises the question of whether VR also alters the FOG phenomenon. We investigate the effects of naturalistic VR on gait and FOG characteristics in PD patients. Patients walked in a real-world environment and its VR replica under conditions that provoke FOG. Spatiotemporal gait parameters and FOG episodes were compared between environments. Results revealed that a detailed VR replica and large walking area reduced the effect of VR on gait parameters compared to previous reports. Additionally, FOG was provoked by similar triggers with comparable frequency, suggesting VR effectively replicates FOG heterogeneity. This work demonstrates the feasibility of VR to study gait and FOG in PD and informs future VR applications.

## Introduction

Freezing of gait (FOG), defined as “a brief, episodic absence or marked reduction of forward progression of the feet despite the intention to walk,”^1^ is one of the most debilitating symptoms of Parkinson’s disease (PD). It is a major cause of falls, loss of independence, and reduced quality of life^2,3^. Development of effective therapies has been hindered by a poor understanding of FOG pathophysiology. A major barrier to progress in the field is the challenge of eliciting FOG episodes in traditional laboratory settings, as FOG episodes are unpredictable and often triggered by complex, real-life environmental demands^4–6^. Common FOG triggers include performing turns, passing through doorways, navigating crowded spaces, multitasking, and experiencing stress^7,8^. There is considerable heterogeneity in patients’ susceptibility to different freezing triggers, leading to the proposal that multiple pathophysiological mechanisms may exist^9–11^. Therefore, the ability to elicit freezing reliably and with diverse triggers is essential to study the transient changes in sensorimotor processing that lead to FOG episodes, and to assess therapeutic strategies.

Immersive virtual reality (VR) during overground walking has become an effective method for enhancing the ecological validity of brain research in humans, including the study of gait^12–16^. The ability to systematically control the environment with VR to create ecological, patient-specific triggers could provide a powerful method to reliably elicit freezing, without being limited to the small set of static triggers that can be implemented in a physical laboratory (e.g., turning in place or straight walking)^5,11,16^. Indeed, a few studies using fully immersed virtual reality during overground walking have shown promise: FOG episodes have been successfully induced in VR, such as simulating walking on an elevated plank^17^ or by varying the width of a virtual doorway^14^. These early studies demonstrate that immersive VR can replicate complex, real-life demands during overground gait, providing a controlled environment to elicit FOG episodes in patients with PD. However, more recent studies using VR to simulate real-world environments have faced challenges in reliably inducing FOG episodes^18–20^. This raises an important question about how similar FOG episodes observed in VR are to those occurring in real-world scenarios. Bridging this gap is key to optimizing VR-based research for understanding and treating FOG.

To study PD gait pathophysiology using VR, it is essential to understand and minimize effects of the VR environment on gait behavior. Multiple studies in both healthy adults^21–25^ and patients with PD^18,19^ have shown significant gait alterations in VR compared to real world. These studies have typically used abstract representations of the real environment with short walkways spanning 6–7 meters^18,19,21–23,25^. Walking in VR is characterized by decreased step length and speed, increased step width, and increased variability of those spatiotemporal measures. These differences are thought to reflect a more cautious or conservative gait driven by factors such as reduced visual cues (e.g., shape, texture, placement, and number of objects affecting depth and distance perception) and diminished sensory feedback about body position in VR^23,24,26–29^. Furthermore, limited physical walking space and uncertainty in VR environments may create self-imposed boundaries, reducing the willingness to walk naturally or explore due to fear of colliding with objects. Given that altered stepping behavior can influence FOG^30^, it is essential to investigate how VR designs can be optimized to promote natural gait patterns and reliably provoke ecological FOG.

This study investigates whether prior findings of altered gait behavior in VR can be mitigated and evaluates the feasibility of using VR to provoke freezing episodes comparable to those observed in the real world. To achieve this, we leverage advancements in VR technology to improve the rendering quality of the virtual environment and expand the walking area, as compared to prior reports, to encourage naturalistic walking behaviors. We directly compared spatiotemporal gait parameters and FOG episodes during multiple freeze-provoking tasks between the real and virtual environments. We hypothesized that these improvements would reduce discrepancies between VR and real-world gait parameters and recapitulate the heterogeneity across patients in freezing severity and susceptibility to specific triggers. Deeper insights into VR’s effects on gait behavior and FOG will inform the design of future VR environments, enabling more effective assessment of FOG and investigation of how diverse environmental demands influence gait dysfunction in PD.

## Results

### Patient characteristics

**Fig. 1:**
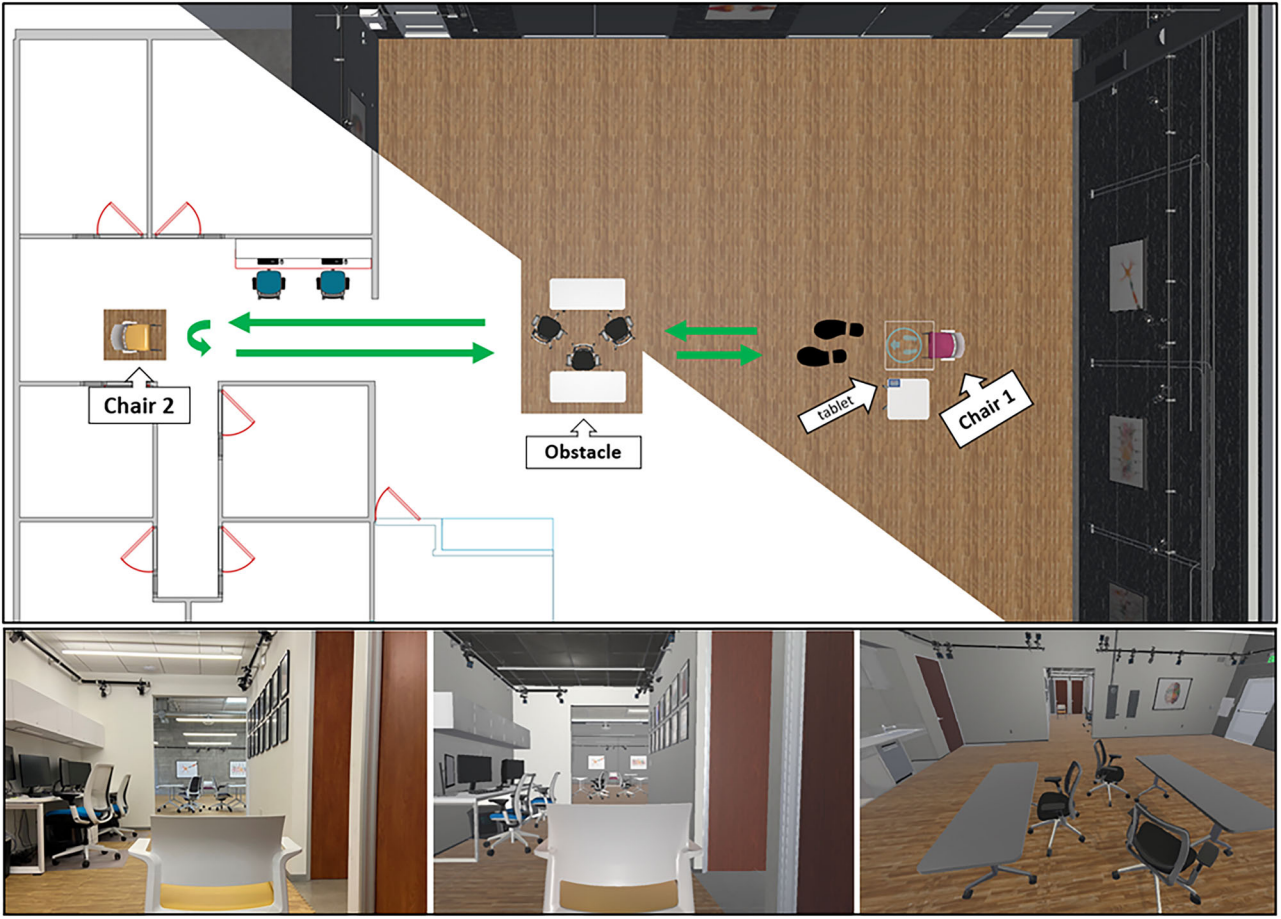
The recording space in real-world and virtual reality (VR) environments. (Top) A top-down view of the lab space layout side-to-side with the virtual replica. Green arrows represent the walking path. For a given trial, participants walk from Chair 1 to Chair 2 and back to Chair 1. The obstacle shown was only present during the obstacle condition. The tablet shown is used in the hurry condition, where participants tap on it to turn the alarm on and off. Detailed instructions for the conditions are provided in the Methods section. (Bottom Left) A view from Chair 2 to Chair 1 in real-world. (Bottom Middle) A view from Chair 2 to Chair 1 in VR. (Bottom Right) VR representation of the arrangement of tables and chairs in the obstacle condition. Note that the quality of this image differs from that viewed through the headset due to the two separate images projected to each lens, as well as the resolution and image curvature from the lenses.

Sixteen patients with PD and a self-reported history of FOG participated in the study, which involved walking along a designated path in both a real-world environment and its VR replica (Fig. 1). Patient demographics and clinical characteristics are presented on Table 1. They completed four randomized and counterbalanced walking conditions carefully matched across environments: 1) normal walking (baseline), 2) walking while performing a digit monitoring task^31^ (dual task), 3) walking under time constraints by activating and deactivating an alarm (hurry), and 4) navigating between tables and chairs placed along the path (obstacle). Patients remained on their regular dopaminergic medication schedule throughout the study.

**Table 1:** Participant Characteristics

Patient #	Sex	Age	Years since PD diagnosis	MOCA	PAS	UPDRS III	H&Y	ABC (%)	NFOG Total	FOG > 1 per day	Froze during study	Trials completed VR | RW
1	M	65	8	28	12	32	2.5	53.25	19	Y	Y	16 | 16
2	M	64	10	23	24	–	2.5	72.31	16	N	N	16 | 16
3	M	69	11	30	0	–	2	78	9	N	N	16 | 16
4	M	72	11	16	15	–	2.5	63.12	16	N	N	12 | 16*
5	F	59	10	22	10	15	2.5	87.5	18	Y	Y	16 | 16
6	M	73	20	24	11	40	4	59.69	17	Y	Y	8 | 8
7	F	67	16	29	7	21	2.5	65.75	21	Y	N	16 | 16
8	M	64	8	25	17	27	3	62.5	19	Y	Y	16 | 16
9	M	70	15	26	12	28	3	40	23	Y	Y	16 | 16
10	F	63	26	23	13	43	3	42.5	21	Y	Y	16 | 16
11	M	77	8	26	9	29	3	81.88	14	Y	Y	16 | 16
12	F	53	19	27	7	28	3	55	19	Y	Y	16 | 16
13	M	73	9	24	8	21	2.5	73.13	21	Y	Y	12 | 12
14	M	62	6	25	24	30	2.5	81.87	22	Y	Y	16 | 16
15	F	57	16	27	12	17	3	28.75	24	Y	Y	16 | 16
16	F	59	5	25	31	70	3	19.06	28	Y	Y	8 | 8

*M* Male, *F* Female, *MOCA* Montreal Cognitive Assessment, *PAS* Parkinson Anxiety Scale, *UPDRS* Unified Parkinson’s Disease Rating Scale, *H&Y* Modified Hoehn and Yahr, *ABC* Activities-specific Balance Confidence Scale, *VR* virtual reality, *RW* real world. UPDRS was not available in 3 patients. *Participant 4 completed 16 real-world trials but 12 VR trials due to fatigue; only 12 trials from each environment were analyzed to ensure a balanced comparison.

### Impact of task condition on spatiotemporal gait parameters is similar in real and virtual environments

**Fig. 2:**
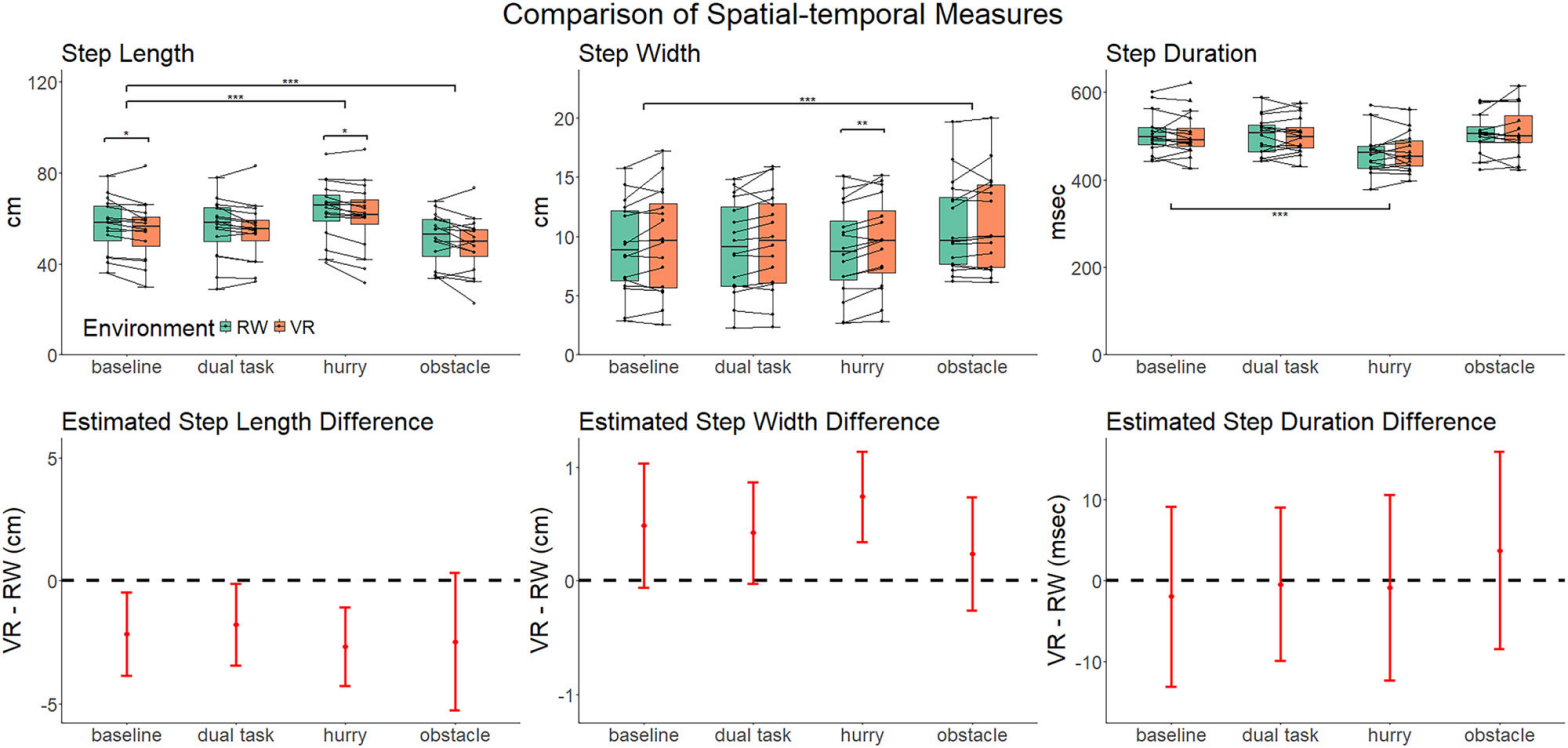
Environment and condition effects on spatiotemporal gait parameters. The top row shows the boxplot and individual participant means (lines) of step length (left), step width (middle), and step duration (right) in each condition for virtual reality (VR) and real world (RW) environments. The bottom row shows the estimated mean difference with a 95% confidence interval between the two environments at each condition. The black dotted line represents a difference of zero (null). Significance is represented by *p *<* 0.05, **p *<* 0.01, ***p *<* 0.001, adjusted with FDR.

Spatiotemporal gait parameters were calculated from 3D optical motion capture data during straight walking segments. Separate linear mixed effects models were used to analyze the effects of environment and task condition on step length, step width, and step duration. Significant condition effects were observed for all spatiotemporal gait measures. There were no condition-by-environment interactions, suggesting that the effects of condition were consistent across environments (Fig. 2). Therefore, we performed pairwise comparisons of the effect of each of the condition relative to the baseline collapsed across environments to examine the impact of condition on gait. Relative to the baseline condition, the hurry condition increased step length by 6.2 cm (standard error (SE) = 1.25, *p* < 0.001) and decreased step duration by 40.88 milliseconds (SE = 7.80, *p* < 0.001). The obstacle condition decreased step length by 6.36 cm (SE = 0.59, *p* < 0.001) and increased step width by 1.58 cm (SE = 0.30, *p* < 0.001). Detailed model summaries and condition comparisons for spatiotemporal measures are presented in Supplementary Tables 2 to 6.

**Fig. 3:**
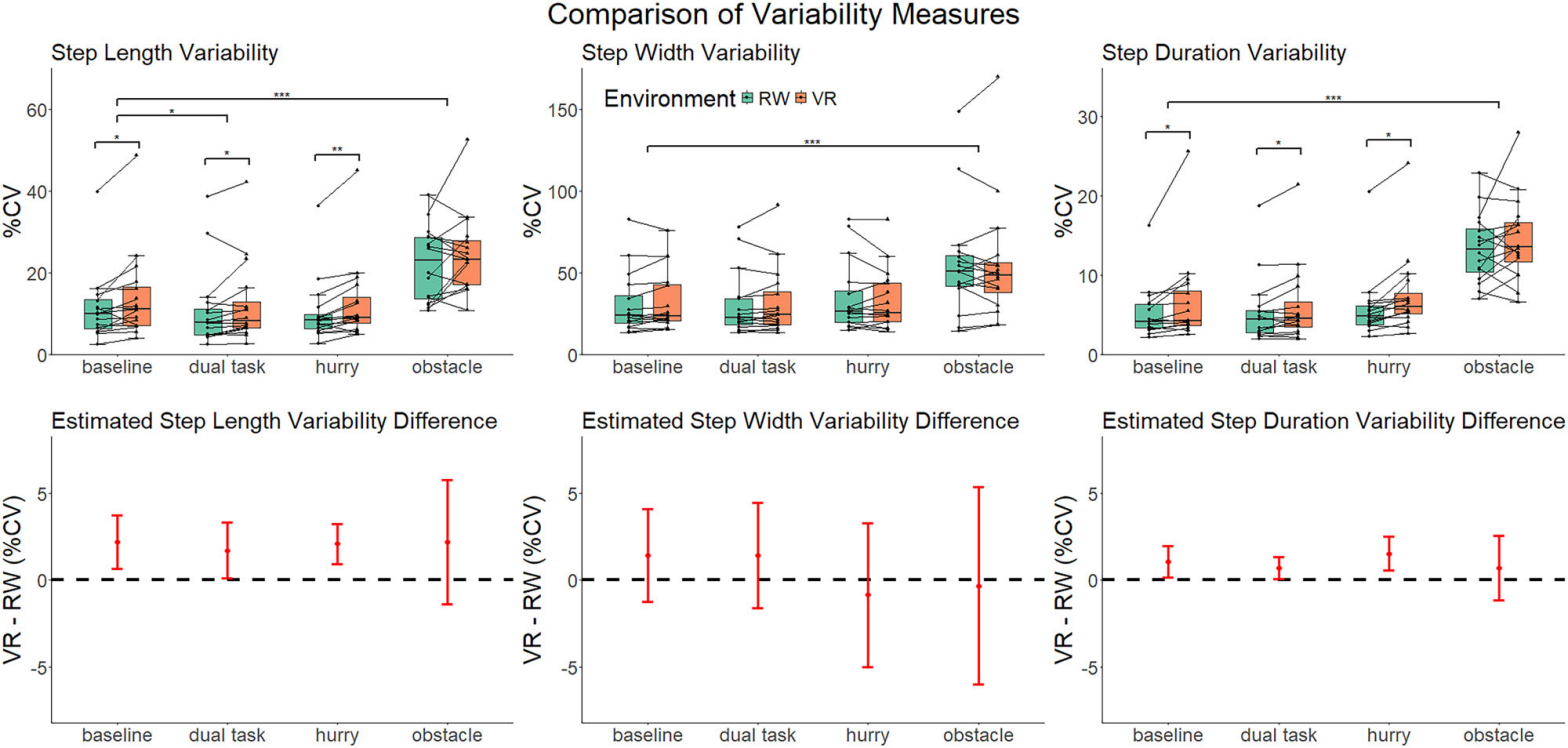
Environment and condition effects on spatiotemporal gait variability. The top row shows the boxplot and individual participant percentage coefficient of variation (%CV) of step length (left), step width (middle), and step duration (right) at each condition for virtual reality (VR) and real world (RW) environments. The bottom row shows the estimated differences with a 95% confidence interval between the two environments at each condition. The black dotted line represents a difference of zero (null). Significance is represented by *p *<* 0.05, **p *<* 0.01, ***p *<* 0.001, adjusted with FDR.

In a similar analysis of gait variability, linear mixed models on the coefficient of variation (%CV) of each spatiotemporal gait parameter (i.e., step length %CV, step width %CV, and step duration %CV) also revealed significant main effects of condition but no condition-by-environment interactions (Fig. 3). Relative to the baseline condition, the obstacle condition increased step length %CV by 11.24% (SE = 1.46, *p* < 0.001), increased step width %CV by 25.50% (SE = 4.81, *p* < 0.001), and increased step duration %CV by 8.40% (SE = 0.79, *p* < 0.001). The dual task condition decreased step length CV by 1.37% (SE = 0.56, *p* = 0.042). Detailed model summaries and condition comparisons for variability measures are presented in Supplementary Tables 7 to 11.

### VR has a small but statistically significant impact on some spatiotemporal gait parameters

Significant effects of environment were observed in step length for baseline and hurry conditions, and in step width for the hurry condition (Fig. 2, bottom). Relative to the real-world environment, VR reduced step length by 2.16 cm (SE = 0.79, *p* = 0.03) in baseline and 2.68 cm (SE = 0.75, *p* = 0.01) in hurry. VR increased step width by 0.74 cm (SE = 0.19, *p* = 0.005) in the hurry condition. There were no interactions between condition and environment. Supplementary Table 12 provides full details on the estimated effects, confidence intervals, and *p*-values for all the planned pairwise contrasts.

VR also increased gait variability, with significant environment effects observed in step length %CV and in step duration %CV during baseline, hurry, and dual task conditions (Fig. 3, bottom). Relative to the real world, VR increased step length %CV by 2.19% in baseline (SE = 0.72, *p* = 0.02), 2.08% in hurry (SE = 0.54, *p* = 0.01), and 1.72% in dual task (SE = 0.74, *p* = 0.048). VR increased step duration %CV by 1.07% in baseline (SE = 0.43, *p* = 0.043), 1.53% in hurry (SE = 0.45, *p* = 0.018), and 0.70% in dual task (SE = 0.30, *p* = 0.043). Supplementary Table 13 provides full details on the unstandardized effect sizes, confidence intervals, and p-values for all the planned contrasts.

### VR impacts freezing duration but not the provoking triggers

There were 271 FOG episodes occurring in 12 of the 16 patients (117 in real-world and 154 in VR). The median number of freezing episodes per participant among those who froze was 17 (IQR 6–34.25, range 1–78). Freezing was observed during 60% of turns, (31% with a secondary cognitive task, 29% without), 26% of obstacles, 5% of gait initiations (start hesitations), and 4% of straight walking segments. The median duration of a freezing episode was 2.6 s (IQR 1.2–6.9 s, range 0.3–80 s). Nearly all participants who froze during the study did so in both real and virtual environments (10/12 patients). The two patients who froze exclusively while walking in VR had only 1 and 3 freezing episodes total.

**Fig. 4:**
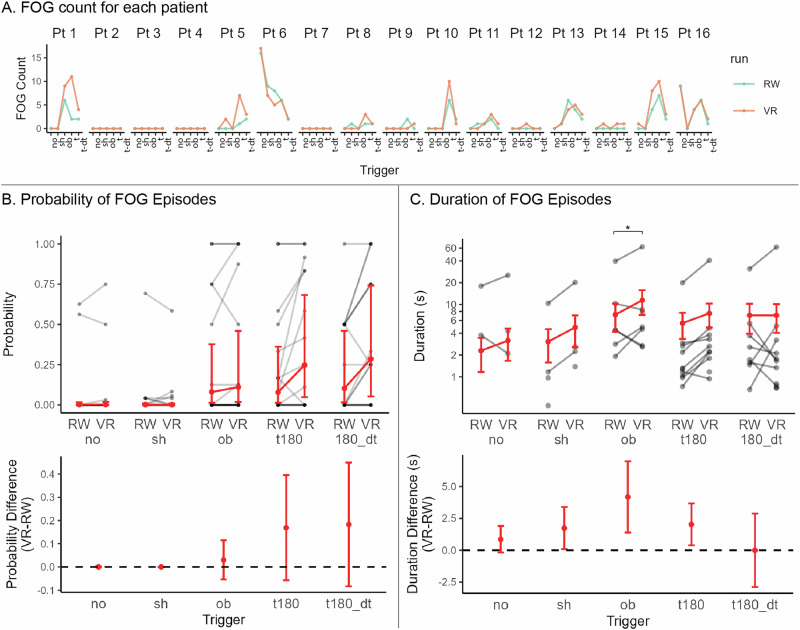
Environment and condition effects on FOG. **A** Number of FOG episodes for each individual participant (Pt) and trigger in real-world (RW) and virtual reality (VR) shows strong concordance in trigger susceptibility between environments. **B** Top plot shows individual participant freezing probabilities (black lines) and estimated mean with 95% confidence interval (red) for each trigger and environment. Value of 1 represents freezing at every encounter with that trigger type and 0 represents never freezing with that trigger type encounter. Bottom plot shows estimated differences with 95% confidence interval between the two environments within each trigger. **C** Top plot shows individual participant mean freeze duration (black lines) and estimated mean with 95% confidence interval (red) for each trigger and condition. Duration is plotted on log scale to better visualize wide range of values across participants. Bottom plot shows estimated differences with 95% confidence interval between the two environments within each trigger. The black dotted line represents a difference of zero (null). The following abbreviations are used to describe the triggers: (no) – straight walking with no apparent trigger; (sh) – start hesitation; (ob) – freezing observed while entering or navigating between tables and chairs in the obstacle condition; (t180) – turns without a dual task; and (t180_dt) – turns with dual task. Significance is represented by *p < 0.05, adjusted with FDR.

Each individual exhibited freezing in response to similar triggers across the two environments, despite variability among patients in the number of episodes and the specific triggers that provoked their freezing (Fig. 4A). This is evidenced by substantial agreement in the occurrence of freezing between environments for each trigger and patient (Cohen’s kappa = 0.74, *p* < 0.001). To assess freezing severity, we estimated effects of environment and triggers on freezing probability and duration using a zero-inflated lognormal linear model of the observed freezing episodes. Planned contrasts in the estimated difference in freezing probability between VR versus and real-world did not reach significance for any triggers (Fig. 4B bottom). However, the freezing episodes were significantly longer in VR compared to real-world for obstacles (difference of 4.18 seconds, SE = 1.4, *p* = 0.03), after adjusting for multiple comparisons (Fig. 4C, bottom). Altogether these results demonstrate that the susceptibility to freezing in response to a given trigger type is similar in real and virtual environments, but freezing events may last longer in VR depending on the trigger. Detailed model summary and planned contrasts are presented in Supplementary Tables 14 and 15.

## Discussion

We examined spatiotemporal gait and FOG characteristics during multiple freeze-provoking tasks performed in real-world and VR environments to assess the impact of immersive VR on gait and FOG during overground walking. Our results extend prior work by minimizing the effect of VR on spatiotemporal gait parameters and by demonstrating that multiple freeze-provoking tasks can provoke freezing similarly in real and virtual environments. Individual participant’s FOG episodes were generally similar in real and virtual environments in terms of their eliciting trigger types and frequency, but freezing episodes were slightly longer in VR for some triggers.

Recent studies comparing overground gait in real-world and virtual environments delivered via head-mounted display (HMD) demonstrated decreased step length and velocity, increased step width, and increased variability of those spatiotemporal measures during straight walking^18,19,22^. Here, PD patients also exhibited shorter step length and higher step length variability in the baseline condition (i.e., straight walking) in VR, but the effects were considerably smaller. Prior studies have reported a mean decrease of 5 cm in step length in healthy participants^32^, and 6 cm in PD patients when walking in VR^19^. In comparison, our study showed a mean of 2.16 cm decrease in step length during the baseline condition, highlighting that VR effects on gait can be attenuated.

We analyzed several hundred steps per patient, making our design sensitive to small changes in gait parameters which may or may not be clinically meaningful. Baudendistel et al. (2024) reported MCID in PD patients based on the triangulation of multiple clinical measures. They identified MCIDs for four gait parameters during straight walking: step velocity at 8.2 cm/s, step length at 3.6 cm, step length %CV at 0.7%, and step duration %CV at 0.67%^33^. Our results during straight walking (baseline condition) show that, on average, the estimated differences in step length (2.16 cm) and velocity (3.23 cm/s) between the real-world and VR are less than the reported MCID, although step length variability (2.19%CV) and step duration variability (1.07%CV) exceed the MCID. While uncertainty in VR may contribute to increased spatiotemporal variability, the absence of clinically significant changes in step length and velocity on average suggests that these measures can be reliably assessed and compared in VR. Moreover, task manipulations like hurry and obstacle conditions resulted in step length and velocity changes far exceeding both the MCID and VR effects, highlighting that VR-induced changes are minimal compared to task- and disease-driven gait alterations. Considering that step length and gait speed are proven quantitative indicators of PD function and progression^34–36^, more work is needed to determine whether the VR effects on step length and velocity can be further normalized.

Creating a naturalistic walking experience in VR can be challenging due to two major factors: 1) limited space for walking when viewing the VR environment, and 2) providing the appropriate sensory feedback when walking in VR^28^. The presence of objects, textures, shadows, and visual feedback of one’s body in VR impacts distance perception, potentially affecting spatiotemporal estimation during gait^37–39^. In our VR setup, we intentionally included environmental features such as chairs, tables, doors, wall art, and textures that matched our real laboratory, which likely increased depth cues for the participants when walking. This contrasts with previous studies involving PD patients in VR, where geometric representations of laboratory spaces were used^18,19^. In addition, we extended the recording space as compared to previous reports with the goal of reducing a sense of confinement. Concerns about colliding with physical objects may limit immersion when walking in VR^40^. Most prior overground VR walking studies limited walk length to approximately 7 meters, with available walk width often below that length due to equipment limitations^18,19,22,23,25^. Having a large recording environment may minimize participants’ concern of collision with objects in the physical environment and, consequently, attenuate cautious gait behavior (i.e., shorter steps, slower speed, and wider steps). A recent study showed that changing the physical width of the walkway in the real world and VR can lead to differences in step cadence^23^. While more studies are needed to investigate the impact of the physical environment on VR walking, we believe that a larger recording space to reduce collision concerns may be important to normalize gait behavior, perhaps especially for PD patients who often have anxiety-related motor symptoms and avoidance behaviors^41^.

Despite the enhanced textures and cues and a larger space, we still observed statistically significant decrease in step length and step length variability. Lower FOV in the horizonal plane has been shown to lead to underestimated distance and movement speed^38,42,43^. The Meta Quest Pro’s horizontal FOV of 106° is smaller than the human eyes, where peripheral vision can extend up to 214° in horizontal diameter^44,45^. In addition, lack of visual feedback of the body in VR may also influence gait behavior, especially in tasks requiring assessment of the body position relative to the environment. Thus, despite efforts to match the VR environment to the real laboratory, perceptual distortion of visual space and optic flow, as well as absence of visual feedback of the body, may have contributed to reduced step length and increased variability in VR. Further investigation is needed to understand the impact of body feedback in VR on gait, as well as to develop methods for easily integrating whole-body visual feedback in VR. These considerations on VR environmental features and physical space for walking, as well as equipment limitations, are critical for future VR implementation in gait studies.

Although we were unable to completely normalize spatiotemporal gait kinematics in VR, several key characteristics of FOG were similar. Our results demonstrate that FOG episodes in VR can be induced by the same challenging situations that trigger freezing in the real world, while also capturing intersubject variability in susceptibility to specific freezing triggers. Furthermore, the probability of freezing upon encountering a particular trigger was not significantly increased in VR compared to real-world suggesting that VR itself did not alter the susceptibility to freezing episodes. On the other hand, the duration of freezing tended to be longer in VR, especially for the obstacle trigger which induced the longest episodes. This suggests that walking in a VR environment may have a greater impact on the ability to overcome a freezing episode than on the factors influencing the onset of freezing. Patients use varying strategies to overcome freezing episodes including using visual cues in the environment, adjusting arousal (including both alerting or calming techniques), or changing the pattern of movement such as by lifting the knees high or walking sideways^46^. These strategies may be hindered depending on the VR design, for example due to a paucity of visual detail, differences in emotional responses in VR, and absence of visual feedback of the body.

Although we demonstrate that VR can successfully recapitulate the heterogeneity of FOG in multiple tasks, more research is needed to fully understand how various factors affect FOG and gait outcomes in VR settings. For example, prior work has shown that physiological responses may vary between VR and real-world environments^47,48^ and anxiety and arousal are known to affect FOG^17,41,49^. In our study, the time-pressure condition elicited few freezing episodes, and we did not measure participants’ physiological or emotional responses. Similarly, our dual-task condition did not have the expected detrimental effect on gait or freezing, potentially because patients prioritized gait, or the auditory stimuli acted as rhythmic cues. Nonetheless, the impact of dual task condition was consistent across environments and thus does not detract from our main conclusions. Another limitation is that patients were studied on their usual medication schedules to better represent their daily routines. Thus, freezing episodes may reflect either “on” or “wearing-off” freezing, depending on dose timing and disease status. Medication timing could contribute to potential differences in gait and freezing between tasks for individual patients, but we minimized the impact of this across the group by counterbalancing task order. Future research focused on examining the impact of cognitive and emotional influences, as well as factors such as disease severity and medication status, will further inform the utility of VR in understanding FOG pathophysiology and in developing targeted treatments.

The current work has notable implications for both research and clinical applications in FOG. The difficulty of provoking freezing in the laboratory and heterogeneity of FOG triggers underscores the need for experimental environments and tasks that can be adapted to individual patients^7,11^. By harnessing the power of VR to manipulate visual perception, we can simulate real-world challenges that contribute to FOG. For example, the current findings demonstrate that presenting a visually cluttered environment in VR can effectively replicate the freezing episodes patients experience in the real world. This approach offers a scalable and flexible method for creating context-specific FOG triggers by dynamically modifying spatial constraints, such as altering the number of distractors and obstacles along a path or adjusting the narrowness of walking spaces. Importantly, these changes can be made on the fly and without the need to physically alter the real environment. From a research perspective, this offers the opportunities to understand the effect of specific environmental manipulations on freezing behavior and associated biophysical or neural signals. In addition, as treatment and rehabilitation strategies targeting FOG continue to advance, this approach lays the groundwork for developing personalized, adaptable VR-based interventions and assessments that more effectively provoke freezing and reflect the challenges patients face in daily life.

In conclusion, our findings demonstrate that VR can provoke FOG in PD patients with a history of FOG, and that the effects of being hurried, performing dual tasks, and navigating obstacles on spatiotemporal gait parameters and FOG are comparable between real and virtual environments. While significant differences in some spatiotemporal gait parameters remain between VR and the real world, enhancing the visual detail of the VR environments and providing larger walking spaces have mitigated previously reported effects of VR on gait. These factors should be considered in future VR design. VR shows promise to provoke ecological FOG, which is essential to study FOG pathophysiology and develop personalized assessments and treatments.

## Methods

### Participants

Sixteen patients with PD and self-reported history of FOG participated in the study. Inclusion criteria were diagnosis of PD by a movement disorders neurologist according to consensus criteria^50^, self-reported FOG in the past month (yes to question 1 on the new freezing of gait questionnaire^51^, ability to walk without any assistive device, normal or corrected-to-normal vision, and no other neurological conditions or problems affecting their ability to walk independently. All participants provided informed consent, and the study was approved by University of California, Los Angeles, Institutional Review Board (#22-001462). Participants took their typical PD medication dose and frequency during the day of the study. Disease severity and symptoms known to be associated with freezing of gait were assessed prior to the experiment with the following assessments: Montreal Cognitive Assessment (MOCA, cognition)^52^, Parkinson Anxiety Scale (PAS)^53^, Unified Parkinson’s disease rating scale part 3 (UPDRS III, motor symptom severity)^54^, Modified Hoehn and Yahr (H&Y), and Activities-specific Balance Confidence Scale (ABC)^55^. Demographic and clinical data are presented in Table 1.

### Task

The study took place in a motion capture recording space made up of a main room and a hallway (Fig. 1). Participants walked along a pre-determined straight path 16 meters long: 11 meters in the main room and 5 meters in the hallway. A single trial involved walking from one end of the path to the other, turning 180 degrees, and walking back to the starting point. Two chairs marked the two ends of the walking path. The width of the main room was 13.2 meters and the width available for walking in the hallway was 2 meters (from the wall to the chairs where experimenters were sitting). Participants performed the following four tasks to provoke FOG while walking along the pre-determined path:

A. *Baseline* – Participants were instructed to make a voluntary stop at any point before the 180-turn, and again after the turn, to elicit start hesitations.
B. *Obstacle* – Participants navigated between tables and chairs placed in the middle of the walking path (Fig. 1, bottom row).
C. *Hurry* – Participants walked through the big room under time pressure created by mimicking a home alarm exit/entry delay scenario. At the start of the trial, they tapped a tablet depicting an alarm display, which was placed at the corner of a table near the starting point. This initiated a countdown conveyed by a beeping sound that progressively increased in volume and rate. A noxious siren sounded if the participant did not reach the hallway before the end of the countdown. Upon re-entering the main room after the 180-degree turn, the countdown beep started again representing the entry delay. Tapping the tablet near the starting point prior to the deadline prevented the noxious alarm. The countdown duration was determined for each participant prior to the experiment. To elicit a sense of time pressure, it was set to 1 second faster than the duration it took to walk the same distance at a comfortable pace. This pace is calculated prior to the start of the study when participants walked the entire length of the pathway.
D. *Dual task* – Participants performed a digit monitoring task while walking^31^. An auditory stream of random numbers was played at a rate of one per second throughout the trial, either from the head-mounted display in VR, or by a speaker in real-world. Participants were instructed to silently count the number of times two specific digits were presented and to report the total at the end of each trial. The two digits were randomly assigned verbally by the physical therapist walking with the participant at the beginning of each trial. Participants were encouraged to track the digits to the best of their abilities, but feedback on the accuracy was not provided.

Participants performed the four walking tasks while wearing a wireless HMD (Meta Quest Pro, Reality Labs, Meta Platforms, Menlo Park, CA) and without the HMD. The HMD projected a virtual environment that carefully replicated the real recording space (Fig. 1). The HMD offers a resolution of 1800 × 1920 pixels per eye, a refresh rate of up to 90 Hz, and a field of view of 106° horizontally and 96° vertically. To ensure high fidelity in VR, we streamed the VR environment to the headset via Meta Link on a high-performance Windows computer equipped with a 12th Gen Intel® Core™ i9-12900KF (16-core, 3.19 GHz), 64 GB of RAM, and an NVIDIA GeForce RTX 3090. The VR environment and tasks were built using Unity (Version 2021.3.13f1, Unity Technologies, San Francisco, CA).

Before the experiment, verbal instructions were provided for each walking task. After each instruction, participants performed the task once in the real-world environment to demonstrate comprehension. During the experiment, the order of environments (VR or real-world) was counterbalanced across participants. The walking tasks were performed in blocks of four trials, one of each walking task. The order of the tasks within each block was randomized across blocks and participants. However, for each participant, the trial order was the same in both environments to ensure within-subject differences in gait features across environments did not reflect differences in task order.

Participants performed one practice block followed by four experimental blocks (16 total experimental trials) in each environment. During the practice block, participants were specifically told that the block would not be counted towards the study. Participants were encouraged to explore the environment and walk beyond the designated path for the baseline task during the practice block in the VR environment to maximize comfort in the VR environment. Only the experimental blocks were used for statistical analysis. Four participants completed fewer blocks due to fatigue (see Table 1). Participants were supervised by a physical therapist throughout the study but received no assistance during FOG episodes unless deemed necessary by the physical therapist to prevent loss of balance.

### Kinematic data acquisition and processing

Kinematic data were collected using 43 motion capture cameras with a sample rate of 120 Hz (Optitrack, NaturalPoint, Inc., Corvallis, OR). Two of those cameras were used as video cameras (instead of motion capture) to film FOG episodes. Velcro straps and reflective markers were placed on the participants according to the plug-and-play Conventional Lower skeleton marker set from Optitrack Motive software. Marker placements included the left and right anterior superior iliac spines, posterior superior iliac spines, lateral epicondyle of femurs, midline of the thighs, midline of the shins, lateral malleolus of the fibulas, and second distal phalanx of the feet. Participants walked in their own sneakers, and thus the second distal phalanx marker was placed over the participants shoes after palpation.

Kinematic data were processed with custom scripts in MATLAB. Marker data was low-pass filtered offline at 12 Hz. Heel strike events were identified based on kinematic methods tested in clinical populations^56,57^. After using the algorithms to identify heel strikes, visual inspection to identify false positive and missing heel strikes was performed by two researchers (BY, LM). Discrepancies were discussed between the researchers until a consensus was reached. The variables of interest were step length, width and duration at each step, and their variability expressed as percentage coefficient of variation (%CV, calculated across steps in each trial). We focused on these variables because they are commonly reported for meta-analyses and minimal clinically important differences (MCID) are available in PD^33,58,59^. The first step after gait initiation and before and after voluntary stops, turns, and FOG episodes, as well as the steps falling within FOG episodes, were excluded from analyses.

*Step length*, measured in centimeters (cm) was defined as the distance between the ipsilateral heel marker of the current heel strike to the contralateral heel marker of the previous heel strike, in the direction of the forward movement. Since participants did not walk perfectly parallel with the axes of the recording space and could deviate from their path (e.g., during obstacle condition), the anterior-posterior axis was defined by the vector of the hip centroid displacement from the previous step to the current step (i.e., direction of the step). In other words, step length is the scalar projection of the vector norm between the right and left heel markers onto the vector of the hip centroid from the previous step to the current step. The orthogonal of that scalar projection was considered *step width*. *Step duration* is the time in milliseconds from the previous to the current heel strike. We also calculated and modeled step velocity as an indicator of gait velocity. Gait velocity is a common spatiotemporal variable reported in the literature, but since step velocity is a direct scaling of step length divided by step duration, we present the velocity outcomes in the supplementary material only to reduce redundancy.

#### Freezing of Gait identification and trigger classification

The number and duration of FOG episodes were determined based on video annotation of each walking trial by two expert raters (KC, LM) following recent published guidelines^60,61^. To explore whether VR successfully recapitulates real-world FOG heterogeneity, we examined whether individual participants froze in the same situations and with similar severity (frequency and duration) across real and VR environments. For statistical modeling, FOG triggers were classified into the following categories: 1) 180-degree turns without dual task; 2) 180-degree turns with dual task; 3) start hesitations; 4) obstacle (freezing observed while entering or navigating between tables and chairs in the obstacle condition); and 5) straight walking when no apparent trigger was present. Straight walking and start hesitation episodes were rare and therefore were not stratified by task condition (dual task or hurry). The annotation between the two raters were compared using MATLAB functions from the FOGtool^60^. The positive agreement between the raters was 0.92 and the negative agreement 0.98, with a prevalence index of −0.54. Additional agreement parameters can be found on Supplementary Table 1.

#### Statistical analysis

We used linear mixed modeling to evaluate the effect of the environment and task condition on spatiotemporal gait parameters. Linear mixed models have the advantage of accounting for subject-level variability and imbalanced data (i.e., varying number of steps per trial and trials per participant), in contrast to traditional ANOVAs with aggregated data^62–64^.

Separate models were constructed for each spatiotemporal gait measure, with environment (VR, real-world), task condition (baseline, obstacle, hurry, dual task), and their interaction included as fixed effects. To account for individual differences in baseline gait and responses to the VR environment, we included random intercepts and random slopes for environment by participant. No additional covariates were included. When the model did not converge, we used a random intercept-only model; this was necessary for step length %CV and step duration %CV. Planned contrasts assessed the effect of the environment on gait by comparing real-world to VR within each of the four conditions. Reference levels were set to real-world for environment and baseline for task condition.

The spatiotemporal models were built using the lmerTest package^65^, an extension of lme4 in R Studio (2023.12.1 Build 402). Each model was inspected for linearity, homoscedasticity, and normality of residuals. To deal with observed heteroscedasticity in the models, standard errors and critical values from the models and contrasts were adjusted using the clubSandwich package, with the ‘CR2’ estimator for small sample corrections. This approach addresses the bias heteroscedasticity introduces in standard error estimations and resultant inflated Type I error^66^. We applied Satterthwaite’s method to estimate degrees of freedom and generated p-values for the contrasts. Estimated marginal means (unstandardized effect size) from the models and contrasts are presented with standard errors (SE). The significance level was set at 0.05. Reported p-values for the planned contrasts are adjusted for multiple comparisons using the False Discovery Rate (FDR) method. Supplementary tables provide corrected and uncorrected p-values.

To determine whether patients’ freezing episodes were provoked by similar triggers in VR and real-world, we used Cohen’s kappa to quantify the agreement between environments. A code of 1 or 0 was assigned for each trigger, environment, and patient indicating the presence or absence of freezing episodes (16 patients x 5 triggers x 2 environments). Agreement was calculated between environments, such that a high agreement indicates that the presence of freezing for a given patient and trigger is similar across environments. The null hypothesis of no correspondence was assessed by comparing the observed kappa with a null distribution of kappa values constructed by permuting the freezing value across triggers within patients 10,000 times.

To evaluate the effect of the environment and freezing triggers on freezing severity (i.e., FOG probability and duration), we used a zero-inflated lognormal linear model of freeze duration for every encounter with a freezing trigger (duration = 0 when no freeze occurs during that encounter). This modeling approach allows us to account for the high number of zeros in the data (e.g., not every encounter led to a freeze and not every patient froze) and the lognormal distribution of the observed duration when patients were freezing.

In this two-part model, first a mixed effects logistic regression models the probability of not freezing (zero-inflation part of the model). This part treats the response as a binary outcome (freeze of any duration versus no freeze). Then non-zero durations are modeled with a mixed effects generalized linear model with a logarithm link function, which ensures that predicted durations will be positive, and assuming a normal distribution for the errors.

We included environment, trigger types, and their interaction as fixed effects. Participant was included as a random intercept in both parts of the model to account for individual variability in freezing probability and duration. Given our focus on the effects of VR versus the real world on FOG, we conducted planned contrasts to evaluate environmental differences in freezing probability and duration for each trigger based on the model estimates. To facilitate the interpretation of model estimates in the plots and contrasts, the coefficients of probability of *not* freezing from the model output were inverted to show the probability of freezing and logarithm duration were transformed back to its exponential form. The corresponding standard errors were estimated using the delta method. *P*-values were adjusted for all planned contrast using FDR in the model. We used the glmmTMB package in RStudio for the zero-inflated log-normal model^67^.

## Supplementary information

Supplemental Tables

## Data Availability

The datasets used during the current study are available from the corresponding author on reasonable request due to privacy/ethical considerations.
