# Supplementary material for: Effects of virtual reality on spatiotemporal gait parameters and freezing of gait in Parkinson’s disease
Source: NPJ Parkinsons Dis. 2025 Jun 4;11:148. doi: 10.1038/s41531-025-01017-9 (PMC12137916; doi:10.1038/s41531-025-01017-9)
Supplement: Supplementary file 1 — Supplemental Tables [file 41531_2025_1017_MOESM1_ESM.pdf]

Supplementary Information

for

Effects of freeze-provoking scenarios on spatiotemporal gait parameters and freezing episodes in Parkinson's disease: A comparison of real and virtual environments.

Supplementary Table 1. Freezing of gait (FOG) annotation agreement table. 2

Supplementary Table 2. Linear mixed model output for step length. 3

Supplementary Table 3. Linear mixed model output for step width. 4

Supplementary Table 4. Linear mixed model output for step duration. 5

Supplementary Table 5. Linear mixed model output for step velocity. 6

Supplementary Table 6. Pairwise comparisons of average spatiotemporal measures between the baseline and other conditions. 7

Supplementary Table 7. Linear mixed model output for step length variability. 8

Supplementary Table 8. Linear mixed model output for step width variability. 9

Supplementary Table 9. Linear mixed model output for step duration variability. 10

Supplementary Table 10. Linear mixed model output for step velocity variability. 11

Supplementary Table 11. Pairwise comparisons of average spatiotemporal *variability* measures between the baseline and other conditions. 12

Supplementary Table 12. Planned contrast of spatiotemporal measures between environments (virtual reality – real world) per condition. 13

Supplementary Table 13. Planned contrast of spatiotemporal *variability* between environments (virtual reality – real world) per condition. 13

Supplementary Table 14. Two-part logistic-lognormal model for freezing of gait duration and probability. 14

Supplementary Table 15. Planned contrasts of the effect of environment (virtual reality vs. real world) on both freezing probability and freezing duration. 15

Supplementary Table 1. Freezing of gait (FOG) annotation agreement table.

Subject	*Total positive agreement*	*Total negative agreement*	*Total prevalence index*	*Total number FOG by rater1*	*Total number FOG by rater2*	*Total number FOG by rater1 only*	*Total number FOG by rater2 only*	*Total number FOG reached consensus*
**1**	0.799	0.976	-0.791	34	29	5	0	34
**5**	0.884	0.990	-0.841	15	15	1	1	15
**6**	0.964	0.975	-0.179	78	72	6	0	78
**8**	0.882	0.993	-0.894	7	6	1	0	7
**9**	0.771	0.988	-0.904	3	3	0	0	3
**10**	0.918	0.993	-0.845	19	19	0	0	19
**11**	0.856	0.990	-0.874	9	10	0	1	9
**12**	0.998	1.000	-0.652	1	1	0	0	1
**13**	0.858	0.986	-0.817	26	27	0	1	26
**14**	0.902	0.993	-0.871	3	3	0	0	3
**15**	0.788	0.978	-0.811	34	36	0	2	35
**16**	0.707	0.945	-0.682	43	36	8	1	41
**total**	0.921	0.977	-0.543	272	257	21	6	271

Supplementary Table 2. Linear mixed model output for step length.

Model Equation: *Step_Length ~ Environment*Condition + (1 + Environment | subject)*

	Step Length (cm)						
Predictors	*Estimates*	*std. Error*	*CI lower*	*CI upper*	*Statistic*	*df*	*p*
(Intercept)	56.805	2.962	50.492	63.118	19.179	15.000	**0.000**
Environment [VR]	-2.160	0.790	-3.844	-0.475	-2.733	14.984	**0.015**
Condition [hurry]	6.456	1.333	3.598	9.314	4.844	14.055	**0.000**
Condition [dual task]	-0.868	0.981	-2.972	1.235	-0.885	14.076	0.391
Condition [obstacle]	-6.209	0.902	-8.146	-4.271	-6.884	13.776	**0.000**
Environment [VR] × Condition [hurry]	-0.515	0.710	-2.039	1.008	-0.726	14.000	0.480
Environment [VR] × Condition [dual task]	0.380	0.762	-1.253	2.014	0.499	14.066	0.625
Environment [VR] × Condition [obstacle]	-0.304	0.958	-2.361	1.752	-0.318	13.885	0.755
σ^2	80.55						
τ_{00}	140.34						
τ_{11}	7.94						
ρ_{01}	0.29						
ICC	0.66						
N	16						
Observations	20170						
Marginal R^2 / Conditional R^2	0.083 / 0.685						

Abbreviations: CI, 95% confidence interval; df, degree of freedom; p, probability value; σ^2, residual variance; τ_{00}, random intercept variance; τ_{11}, random slope variance; ρ_{01}, random slope-intercept correlation; ICC, intraclass correlation coefficient, N, number of subjects.

Supplementary Table 3. Linear mixed model output for step width.

Model Equation: *Step_Width ~ Environment*Condition + (1 + Environment | subject)*

	Step Width (cm)						
Predictors	*Estimates*	*std. Error*	*CI lower*	*CI upper*	*Statistic*	*df*	*p*
(Intercept)	9.143	0.967	7.082	11.204	9.455	15.000	**0.000**
Environment [VR]	0.489	0.256	-0.058	1.036	1.908	14.927	0.076
Condition [hurry]	-0.341	0.189	-0.747	0.065	-1.800	14.055	0.093
Condition [numbers]	0.004	0.129	-0.272	0.281	0.033	14.076	0.974
Condition [obstacle]	1.709	0.325	1.011	2.408	5.255	13.776	**0.000**
Environment [VR] × Condition [hurry]	0.253	0.246	-0.274	0.779	1.028	14.000	0.321
Environment [VR] × Condition [numbers]	-0.066	0.203	-0.501	0.368	-0.326	14.067	0.749
Environment [VR] × Condition [obstacle]	-0.250	0.151	-0.574	0.073	-1.662	13.886	0.119
σ^2	14.17						
τ_{00}	14.92						
τ_{11}	0.55						
ρ_{01}	0.21						
ICC	0.53						
N	16						
Observations	20170						
Marginal R^2 / Conditional R^2	0.020 / 0.537						

Supplementary Table 4. Linear mixed model output for step duration.

Model Equation: *Step_Duration ~ Environment*Condition + (1 + Environment | subject)*

	**Step Duration (msec)**						
Predictors	*Estimates*	*std. Error*	*CI lower*	*CI upper*	*Statistic*	*df*	*p*
(Intercept)	505.605	11.386	481.336	529.875	44.404	15.000	**0.000**
Environment [VR]	-1.929	5.224	-13.065	9.206	-0.369	14.978	0.717
Condition [hurry]	-41.429	8.821	-60.342	-22.515	-4.696	14.055	**0.000**
Condition [dual task]	-4.016	5.294	-15.365	7.333	-0.759	14.076	0.461
Condition [obstacle]	0.534	4.743	-9.654	10.722	0.113	13.776	0.912
Environment [VR] × Condition [hurry]	1.097	4.774	-9.143	11.337	0.230	14.000	0.822
Environment [VR] × Condition [dual task]	1.513	4.283	-7.669	10.695	0.353	14.066	0.729
Environment [VR] × Condition [obstacle]	5.703	3.540	-1.895	13.301	1.611	13.885	0.130
σ^2	3562.23						
τ_{00}	1641.30						
τ_{11}	294.78						
ρ_{01}	0.02						
ICC	0.34						
N	16						
Observations	20170						
Marginal R^2 / Conditional R^2	0.053 / 0.372						

Supplementary Table 5. Linear mixed model output for step velocity.

Model Equation: *Step_Velocity ~ Environment*Condition + (1 + Environment | subject)*

	Step Velocity (cm/s)						
Predictors	*Estimates*	*std. Error*	*CI lower*	*CI upper*	*Statistic*	*df*	*p*
(Intercept)	113.533	6.445	99.796	127.269	17.617	15.000	**0.000**
Environment [VR]	-3.235	2.359	-8.264	1.794	-1.371	14.994	0.191
Condition [hurry]	25.008	5.013	14.260	35.756	4.988	14.055	**0.000**
Condition [dual task]	-0.727	2.366	-5.799	4.345	-0.307	14.076	0.763
Condition [obstacle]	-12.431	2.081	-16.901	-7.962	-5.974	13.776	**0.000**
Environment [VR] × Condition [hurry]	-1.832	2.212	-6.576	2.912	-0.828	13.999	0.421
Environment [VR] × Condition [dual task]	0.120	1.913	-3.980	4.221	0.063	14.066	0.951
Environment [VR] × Condition [obstacle]	-1.542	1.964	-5.757	2.674	-0.785	13.885	0.446
σ^2	406.1						
τ_{00}	669.27						
τ_{11}	68.39						
ρ_{01}	0.30						
ICC	0.65						
N	16						
Observations	20170						
Marginal R^2 / Conditional R^2	0.134 / 0.701						

Supplementary Table 6. Pairwise comparisons of spatiotemporal measures between the baseline and other conditions.

	Condition Comparisons	*Estimates*	*std. Error*	*CI lower*	*CI upper*	*df*	*p*	*FDR p-adjusted*
**Step Length (cm)**	baseline - hurry	6.199	1.254	3.509	8.888	13.999	**<0.001**	**<0.001**
	baseline - dual task	-0.678	0.736	-2.257	0.9	14.066	0.373	0.373
	baseline - obstacle	-6.361	0.592	-7.633	-5.089	13.885	**<0.001**	**<0.001**
**Step Width (cm)**	baseline - hurry	-0.215	0.192	-0.627	0.197	14	0.282	0.423
	baseline - dual task	-0.029	0.098	-0.238	0.181	14.066	0.773	0.773
	baseline - obstacle	1.584	0.303	0.934	2.235	13.885	**<0.001**	**<0.001**
**Step Duration (msec)**	baseline - hurry	-40.88	7.801	-57.611	-24.149	13.999	**<0.001**	**<0.001**
	baseline - dual task	-3.26	4.396	-12.685	6.165	14.066	0.471	0.518
	baseline - obstacle	3.385	5.103	-7.569	14.338	13.885	0.518	0.518
**Step Length Velocity (cm/s)**	baseline - hurry	24.092	4.569	14.291	33.892	13.999	**<0.001**	**<0.001**
	baseline - dual task	-0.667	1.741	-4.4	3.066	14.066	0.707	0.707
	baseline - obstacle	-13.202	1.564	-16.558	-9.846	13.885	**<0.001**	**<0.001**

Supplementary Table 7. Linear mixed model output for step length variability.

Model Equation: *Step_Length_CV ~ Environment*Condition + (1 | subject)*

	Step Length %CV						
Predictors	*Estimates*	*std. Error*	*CI lower*	*CI upper*	*Statistic*	*df*	*p*
(Intercept)	11.575	2.135	7.023	16.127	5.421	14.996	**0.000**
Environment [VR]	2.192	0.716	0.661	3.724	3.061	14.468	**0.008**
Condition [hurry]	-1.032	0.768	-2.674	0.611	-1.343	14.432	0.200
Condition [dual task]	-1.134	0.823	-2.895	0.626	-1.378	14.432	0.189
Condition [obstacle]	11.248	1.852	7.287	15.210	6.073	14.432	**0.000**
Environment [VR] × Condition [hurry]	-0.110	0.600	-1.393	1.173	-0.183	14.426	0.857
Environment [VR] × Condition [dual task]	-0.476	0.882	-2.363	1.412	-0.539	14.454	0.598
Environment [VR] × Condition [obstacle]	-0.008	1.585	-3.399	3.382	-0.005	14.454	0.996
σ^2	22.22						
τ_{00}	75.58						
τ_{11}							
ρ_{01}							
ICC	0.77						
N	16						
Observations	464						
Marginal R^2 / Conditional R^2	0.227 / 0.824						

Supplementary Table 8. Linear mixed model output for step width variability.

Model Equation: *Step_Width_CV ~ Environment*Condition + (1 + Environment | subject)*

	Step Width %CV						
Predictors	*Estimates*	*std. Error*	*CI lower*	*CI upper*	*Statistic*	*df*	*p*
(Intercept)	30.630	4.787	20.428	40.833	6.399	14.997	**0.000**
Environment [VR]	1.423	1.245	-1.237	4.084	1.143	14.518	0.271
Condition [hurry]	3.583	1.876	-0.429	7.595	1.910	14.432	0.076
Condition [dual task]	-0.437	1.107	-2.804	1.930	-0.395	14.432	0.699
Condition [obstacle]	26.381	4.370	17.035	35.727	6.037	14.432	**0.000**
Environment [VR] × Condition [hurry]	-2.282	2.072	-6.714	2.151	-1.101	14.425	0.289
Environment [VR] × Condition [dual task]	0.005	1.986	-4.242	4.251	0.002	14.453	0.998
Environment [VR] × Condition [obstacle]	-1.755	2.889	-7.933	4.422	-0.608	14.453	0.553
σ^2	122.86						
τ_{00}	517.29						
τ_{11}	5.14						
ρ_{01}	0.13						
ICC	0.81						
N	16						
Observations	464						
Marginal R^2 / Conditional R^2	0.153 / 0.840						

Supplementary Table 9. Linear mixed model output for step duration variability.

Model Equation: *Step_Duration_CV ~ Environment*Condition + (1 | subject)*

	Step Duration %CV						
Predictors	*Estimates*	*std. Error*	*CI lower*	*CI upper*	*Statistic*	*df*	*p*
(Intercept)	5.392	0.902	3.469	7.315	5.977	14.991	**0.000**
Environment [VR]	1.072	0.425	0.162	1.981	2.520	14.468	**0.024**
Condition [hurry]	0.419	0.326	-0.279	1.116	1.283	14.432	0.220
Condition [dual task]	-0.134	0.326	-0.831	0.563	-0.412	14.432	0.686
Condition [obstacle]	8.583	1.008	6.427	10.739	8.515	14.432	**0.000**
Environment [VR] × Condition [hurry]	0.453	0.433	-0.474	1.379	1.045	14.426	0.313
Environment [VR] × Condition [dual task]	-0.369	0.389	-1.202	0.463	-0.948	14.454	0.359
Environment [VR] × Condition [obstacle]	-0.377	0.873	-2.245	1.491	-0.431	14.454	0.673
σ^2	8.15						
τ_{00}	16.67						
τ_{11}							
ρ_{01}							
ICC	0.67						
N	16						
Observations	464						
Marginal R^2 / Conditional R^2	0.349 / 0.786						

Supplementary Table 10. Linear mixed model output for step velocity variability.

Model Equation: *Step_Velocity_CV ~ Environment*Condition + (1 + Environment | subject)*

	Step Velocity %CV						
Predictors	*Estimates*	*std. Error*	*CI lower*	*CI upper*	*Statistic*	*df*	*p*
(Intercept)	12.200	2.043	7.845	16.555	5.971	14.997	**0.000**
Environment [VR]	2.560	1.051	0.316	4.803	2.434	14.838	**0.028**
Condition [hurry]	-0.288	0.766	-1.927	1.352	-0.375	14.432	0.713
Condition [dual task]	-1.054	0.767	-2.694	0.587	-1.374	14.432	0.191
Condition [obstacle]	10.589	1.435	7.520	13.659	7.379	14.432	**0.000**
Environment [VR] × Condition [hurry]	-0.073	0.700	-1.571	1.424	-0.105	14.424	0.918
Environment [VR] × Condition [dual task]	-0.687	0.762	-2.318	0.943	-0.902	14.453	0.382
Environment [VR] × Condition [obstacle]	-0.104	1.484	-3.277	3.070	-0.070	14.453	0.945
σ^2	17.01						
τ_{00}	62.45						
τ_{11}	8.69						
ρ_{01}	0.83						
ICC	0.83						
N	16						
Observations	464						
Marginal R^2 / Conditional R^2	0.194 / 0.867						

Supplementary Table 11. Pairwise comparisons of spatiotemporal *variability* between the baseline and other conditions.

	Condition Comparisons	*Estimates*	*std. Error*	*CI lower*	*CI upper*	*df*	*p*	*FDR p-adjusted*
**Step Length CV (%)**	baseline - hurry	-1.087	0.649	-2.474	0.301	14.426	0.115	0.115
	baseline - dual task	-1.372	0.561	-2.571	-0.173	14.454	**0.028**	**0.042**
	baseline - obstacle	11.244	1.46	8.121	14.367	14.454	**<0.001**	**<0.001**
**Step Width CV (%)**	baseline - hurry	2.442	1.579	-0.936	5.82	14.425	0.144	0.216
	baseline - dual task	-0.435	0.913	-2.387	1.518	14.453	0.641	0.641
	baseline - obstacle	25.503	4.809	15.219	35.787	14.453	**<0.001**	**<0.001**
**Step Duration CV (%)**	baseline - hurry	0.645	0.364	-0.133	1.423	14.426	0.097	0.146
	baseline - dual task	-0.319	0.245	-0.843	0.205	14.454	0.213	0.213
	baseline - obstacle	8.395	0.786	6.714	10.076	14.454	**<0.001**	**<0.001**
**Step Length Velocity CV (%)**	baseline - hurry	-0.324	0.645	-1.703	1.055	14.424	0.623	0.623
	baseline - dual task	-1.397	0.499	-2.465	-0.329	14.453	**0.014**	**0.021**
	baseline - obstacle	10.538	1.202	7.967	13.108	14.453	**<0.001**	**<0.001**

Supplementary Table 12. Planned contrast of spatiotemporal measures between environments (virtual reality – real world) per condition.

	Condition	*Estimates*	*std. Error*	*CI lower*	*CI upper*	*df*	*p*	*FDR p-adjusted*
**Step Length (cm)**	*Baseline*	-2.160	0.790	-3.844	-0.475	14.984	**0.015**	**0.031**
	Hurry	-2.675	0.753	-4.281	-1.069	14.981	**0.003**	**0.012**
	Dual task	-1.779	0.782	-3.447	-0.112	14.988	**0.038**	0.051
	Obstacle	-2.464	1.310	-5.257	0.329	14.986	0.080	0.080
**Step Width (cm)**	*Baseline*	0.489	0.256	-0.058	1.036	14.927	0.076	0.101
	Hurry	0.741	0.187	0.343	1.140	14.917	**0.001**	**0.005**
	Dual task	0.423	0.210	-0.024	0.870	14.943	0.062	0.101
	Obstacle	0.239	0.234	-0.260	0.737	14.931	0.323	0.323
**Step Duration (msec)**	*Baseline*	-1.929	5.224	-13.065	9.206	14.978	0.717	0.927
	Hurry	-0.832	5.390	-12.321	10.657	14.975	0.879	0.927
	Dual task	-0.416	4.443	-9.887	9.054	14.983	0.927	0.927
	Obstacle	3.773	5.717	-8.414	15.960	14.980	0.519	0.927
**Step Velocity (cm/s)**	*Baseline*	-3.235	2.359	-8.264	1.794	14.994	0.191	0.191
	Hurry	-5.067	2.537	-10.475	0.341	14.993	0.064	0.191
	Dual task	-3.114	2.148	-7.694	1.465	14.995	0.168	0.191
	Obstacle	-4.776	3.113	-11.411	1.858	14.995	0.146	0.191

Supplementary Table 13. Planned contrast of spatiotemporal *variability* between environments (virtual reality – real world) per condition.

	Condition	*Estimates*	*std. Error*	*CI lower*	*CI upper*	*df*	*p*	*FDR p-adjusted*
**Step Length (%)**	*Baseline*	2.192	0.716	3.724	0.661	14.468	**0.008**	**0.016**
	Hurry	2.082	0.540	3.239	0.926	14.356	**0.002**	**0.007**
	Dual task	1.717	0.744	3.308	0.125	14.432	**0.036**	**0.048**
	Obstacle	2.184	1.672	5.761	-1.393	14.432	0.212	0.212
**Step Width (%)**	*Baseline*	1.423	1.245	-1.237	4.084	14.518	0.271	0.667
	Hurry	-0.858	1.935	-4.997	3.280	14.419	0.664	0.885
	Dual task	1.428	1.428	-1.625	4.481	14.487	0.334	0.667
	Obstacle	-0.332	2.658	-6.016	5.352	14.487	0.902	0.902
**Step Duration (%)**	*Baseline*	1.072	0.425	0.162	1.981	14.468	**0.024**	**0.043**
	Hurry	1.525	0.452	0.556	2.493	14.356	**0.004**	**0.018**
	Dual task	0.703	0.297	0.068	1.338	14.432	**0.032**	**0.043**
	Obstacle	0.695	0.873	-1.171	2.561	14.432	0.439	0.439
**Step Velocity (%)**	*Baseline*	2.560	1.051	4.803	0.316	14.838	**0.028**	**0.037**
	Hurry	2.486	0.833	0.709	4.263	14.802	**0.009**	**0.037**
	Dual task	1.872	0.711	0.356	3.388	14.827	**0.019**	**0.037**
	Obstacle	2.456	1.622	-1.004	5.916	14.827	0.151	0.151

Supplementary Table 14. Two-part logistic-lognormal model for freezing of gait duration and probability.

Model Equation: *duration ~ run*trigger + (1|subj)*
Count Model is estimating the log-transformed duration of freezing when it occurs. Zero-inflated Model is estimating the probability that duration=0 (i.e., the probability of not freezing).

	duration				
Predictors	*Estimates*	*std. Error*	*CI*	*Statistic*	*p*
**Count Model**					
(Intercept)	0.83	0.25	0.34 – 1.33	3.3	**0.001**
run [VR]	0.32	0.19	-0.04 – 0.68	1.72	0.086
trigger [ob]	1.15	0.18	0.79 – 1.50	6.4	**<0.001**
trigger [sh]	0.29	0.2	-0.11 – 0.68	1.44	0.151
trigger [t180]	0.87	0.18	0.53 – 1.22	4.98	**<0.001**
trigger [t180_dt]	1.12	0.21	0.70 – 1.54	5.25	**<0.001**
run [VR] × trigger [ob]	0.14	0.23	-0.32 – 0.59	0.6	0.551
run [VR] × trigger [sh]	0.13	0.27	-0.40 – 0.66	0.49	0.627
run [VR] × trigger [t180]	0	0.22	-0.44 – 0.43	-0.02	0.983
run [VR] × trigger [t180_dt]	-0.32	0.28	-0.86 – 0.23	-1.15	0.252
(Intercept)	13.16		10.51 – 16.48		
**Zero-Inflated Model**					
(Intercept)	6.19	1.03	4.17 – 8.20	6.01	**<0.001**
run [VR]	-0.2	0.45	-1.09 – 0.68	-0.45	0.652
trigger [ob]	-3.75	0.52	-4.76 – -2.73	-7.26	**<0.001**
trigger [sh]	0.42	0.51	-0.57 – 1.42	0.83	0.406
trigger [t180]	-3.72	0.49	-4.67 – -2.76	-7.65	**<0.001**
trigger [t180_dt]	-4.02	0.59	-5.17 – -2.86	-6.79	**<0.001**
run [VR] × trigger [ob]	-0.15	0.62	-1.36 – 1.06	-0.24	0.807
run [VR] × trigger [sh]	0.27	0.72	-1.14 – 1.68	0.37	0.709
run [VR] × trigger [t180]	-1.15	0.57	-2.26 – -0.04	-2.03	**0.042**
run [VR] × trigger [t180_dt]	-1.05	0.73	-2.48 – 0.38	-1.44	0.149
**Random Effects**					
σ^2	13.16				
$\tau_{00\ subj}$	0.28				
ICC	0.02				
N_{subj}	16				
Observations	2313				
Marginal R^2 / Conditional R^2	0.016 / 0.036				

Supplementary Table 15. Planned contrasts of the effect of environment (virtual reality vs. real world) on both freezing probability and freezing duration.

For probability differences, we inverted the model estimates to represent changes in the likelihood of freezing. For duration differences, we converted the log-transformed estimates to their exponential form. The following abbreviations are used to describe the triggers: (no) – straight walking with no apparent trigger; (sh) – start hesitations; (ob) – freezing observed while entering or navigating between tables and chairs in the obstacle condition; (t180) – turns without a dual task; and (t180_dt) – turns with a dual task. All p-values were adjusted using FDR to account for the 10 pairwise comparisons.

	Condition	*Estimates*	*std. Error*	*CI lower*	*CI upper*	*p*	*FDR p-adjusted*
**Probability Difference (a.u.)**	no	<0.001	0.001	-0.002	0.003	0.680	0.850
	sh	<-0.001	0.001	-0.002	0.001	0.909	0.998
	ob	0.030	0.043	-0.054	0.115	0.481	0.687
	t180	0.169	0.115	-0.057	0.396	0.142	0.285
	t180_dt	0.183	0.136	-0.083	0.450	0.177	0.295
**Duration Difference (s)**	no	0.860	0.530	-0.179	1.899	0.105	0.262
	sh	1.736	0.842	0.085	3.387	**0.039**	0.131
	ob	4.181	1.424	1.390	6.971	**0.003**	**0.033**
	t180	2.026	0.836	0.387	3.665	**0.015**	0.077
	t180_dt	-0.003	1.469	-2.882	2.876	0.998	0.998
